# A Comparative Study of the Efficacy of Platelet-Rich Plasma (PRP) vs. Other Conservative Treatments for Lateral Epicondylitis

**DOI:** 10.7759/cureus.70590

**Published:** 2024-10-01

**Authors:** Naveena H M, Manjunatha R, Ankur Salwan

**Affiliations:** 1 Orthopedics, Subbaiah Medical College and Hospital and Research Centre, Molecular Biology Virology Laboratory, Shivamogga, IND; 2 Orthopedics, M. S. Ramaiah Medical College, Bengaluru, IND; 3 Orthopedic Surgery, Jawaharlal Nehru Medical College, Datta Meghe Institute of Higher Education & Research, Wardha, IND

**Keywords:** conservative management, lateral epicondylitis, pain relief, platelet-rich plasma, prtee score, tennis elbow

## Abstract

Background

Lateral epicondylitis, also known as tennis elbow, is a degenerative condition that affects a significant portion of the adult population, particularly those between the ages of 35 and 55. Conventional treatments, including analgesics, physiotherapy, and bracing, often lead to a high recurrence rate. Platelet-rich plasma (PRP) therapy has emerged as a promising alternative, offering regenerative benefits that may enhance tissue healing. This study evaluates the effectiveness of PRP injections compared to traditional conservative therapies in alleviating pain and improving function in individuals with chronic lateral epicondylitis.

Methods

This prospective observational study was conducted at Father Muller Medical College Hospital from April 2018 to August 2019. A total of 44 patients aged 18-60 years with chronic lateral epicondylitis were randomly assigned to two groups: PRP (n = 22) and conservative treatment (n = 22). The conservative group received a combination of physiotherapy, analgesics, and a counterforce brace. The primary outcome was measured using the Patient-Rated Tennis Elbow Evaluation (PRTEE) score, assessed at baseline, four weeks, eight weeks, three months, and six months. The PRTEE score includes pain and function subscales. Data was analyzed using appropriate statistical methods, and significance was considered at p < 0.05.

Results

Both groups significantly improved pain and function during the first eight weeks. However, the PRP group demonstrated superior long-term outcomes. At three and six months, the PRP group had significantly lower pain scores (26.00 ± 3.55 and 19.55 ± 3.33, respectively, p < 0.001) compared to the conservative group (32.23 ± 3.75 and 33.64 ± 3.63). Similarly, the PRP group showed better functional improvement at six months (PRTEE function subscale: 20.00 ± 3.19 in PRP vs. 30.73 ± 4.11 in conservative, p < 0.001). No complications were reported in either group.

Conclusions

PRP injections provide superior long-term pain relief and functional recovery compared to conservative management in patients with chronic lateral epicondylitis. While conservative treatments are effective in the short term, PRP offers a more durable solution with no reported complications. These findings support the use of PRP as a viable alternative for the long-term management of lateral epicondylitis.

## Introduction

Lateral epicondylitis, commonly known as tennis elbow, is one of the most prevalent musculoskeletal conditions affecting the elbow joint. It is characterized by pain and tenderness over the lateral epicondyle of the humerus, primarily due to repetitive stress or overuse of the forearm extensor muscles, particularly the extensor carpi radialis brevis (ECRB) tendon [1]. This overuse results in microtears and collagen degeneration in the tendon, leading to chronic pain and dysfunction. Lateral epicondylitis affects approximately 1-3% of the general population and is particularly common among individuals between 35 and 55 years of age [2]. While the condition is often referred to as tennis elbow due to its association with racquet sports, it is seen in a wide range of occupations and activities that involve repetitive wrist and forearm movements, such as manual labor, office work, and sports [3]. The pathophysiology of lateral epicondylitis involves tendinosis rather than tendinitis, indicating a degenerative rather than an inflammatory process. Studies have shown that the ECRB tendon undergoes microscopic tears, which lead to abnormal vascularity and disorganized collagen fibers [4]. This chronic degeneration is believed to be the primary cause of pain and dysfunction, making lateral epicondylitis a challenging condition to treat effectively. Diagnosis is typically clinical, based on patient history and physical examination findings such as tenderness over the lateral epicondyle and pain during resisted wrist extension or gripping [5].

Conservative treatment is the first line of management for lateral epicondylitis, and it includes a variety of approaches aimed at reducing pain, improving function, and promoting tendon healing. These treatments often include rest, modification of activity, physical therapy, the use of non-steroidal anti-inflammatory drugs (NSAIDs), and counterforce bracing [6]. Physiotherapy, in particular, focuses on stretching and strengthening exercises to alleviate symptoms. While these methods can provide symptomatic relief, their long-term efficacy is often limited, especially in chronic cases [7]. Moreover, many patients experience recurrent episodes of pain after discontinuing treatment, which indicates the need for more effective therapeutic approaches [8]. Corticosteroid injections have also been used as an adjunct to conservative management, particularly in patients with significant pain. While corticosteroids offer short-term pain relief, their long-term benefits are questionable. Studies have demonstrated that while corticosteroid injections reduce pain in the immediate aftermath of treatment, they do not alter the course of the disease, and some patients may experience a relapse of symptoms within months [9]. Additionally, repeated corticosteroid injections are associated with potential adverse effects such as tendon weakening, which could increase the risk of rupture [10]. This has prompted the search for alternative treatment modalities that provide pain relief and enhance tendon healing and function in the long term.

In recent years, platelet-rich plasma (PRP) therapy has emerged as a promising biological treatment for chronic tendinopathies, including lateral epicondylitis. PRP is an autologous blood product that contains a high concentration of platelets, which are rich in growth factors such as platelet-derived growth factor (PDGF), vascular endothelial growth factor (VEGF), transforming growth factor-beta (TGF-β), and fibroblast growth factor [11]. These growth factors play a crucial role in tissue repair by promoting collagen synthesis, angiogenesis, and cell proliferation [12]. When injected into the affected area, PRP is believed to accelerate the body’s natural healing processes by enhancing tissue regeneration at the site of tendon injury [13]. Several clinical studies have evaluated the efficacy of PRP in treating lateral epicondylitis, with encouraging results. Mishra and Pavelko were among the first to report the positive effects of PRP on chronic tendinosis of the elbow, demonstrating significant improvement in pain and function compared to baseline [14]. Subsequent randomized controlled trials have confirmed these findings, showing that PRP provides better long-term outcomes than corticosteroid injections and conservative treatments [15,16]. For example, Gosens et al. conducted a double-blind, randomized controlled trial comparing PRP to corticosteroids in patients with chronic lateral epicondylitis. They found that PRP led to sustained pain relief and functional improvement for up to two years post-injection [17]. Similar results have been observed in other studies, with PRP consistently showing superior long-term benefits compared to conventional treatments [18].

Despite these promising results, PRP therapy is not without its limitations. The variability in PRP preparation techniques, including differences in platelet concentration and activation methods, can lead to inconsistent clinical outcomes [12]. Moreover, the lack of standardized protocols for PRP administration, as well as the high cost of the procedure, has limited its widespread adoption [19]. Further research is needed to optimize PRP preparation and delivery and to determine the long-term efficacy and safety of this treatment in a larger, more diverse patient population. Given the limitations of current conservative treatments and the emerging evidence supporting PRP therapy, this study aims to compare the efficacy of PRP injections with conventional conservative management in the treatment of chronic lateral epicondylitis. By analyzing both short-term and long-term outcomes, this study seeks to provide a clearer understanding of the potential benefits of PRP in managing this common yet challenging condition.

## Materials and methods

Study design

This study was designed as a prospective observational trial to compare the effectiveness of PRP injections with traditional conservative management approaches in treating chronic lateral epicondylitis. The study was conducted at Father Muller Medical College Hospital, Mangalore, India, over 17 months from April 2018 to August 2019. Ethical approval for the study was obtained from the institutional ethics committee, ensuring that all research adhered to ethical standards for clinical trials. Additionally, written informed consent was obtained from all participants before enrollment in the study, ensuring voluntary participation and comprehension of the study’s objectives and procedures.

Study population

Based on clinical signs and symptoms, the study population consisted of 44 patients diagnosed with chronic lateral epicondylitis. Eligible patients were adults aged 18-60 years with persistent symptoms of lateral epicondylitis for at least six months who had not responded to prior conservative treatments such as physical therapy, NSAIDs, or corticosteroid injections. The inclusion criteria focused on patients who were physically capable of undergoing treatment and willing to participate in a structured follow-up for at least six months. Patients were excluded if they had a history of elbow surgery, concurrent elbow pathologies (e.g., fractures or tendon ruptures), were pregnant, or were likely to be lost to follow-up. The exclusion criteria ensured a homogeneous study population and minimized the risk of confounding factors affecting the outcomes.

Randomization and grouping

Patients meeting the inclusion criteria were randomly assigned into one of two treatment groups using a simple randomization method. A computer-generated random number table was used to allocate participants, ensuring an unbiased group distribution. Group A, consisting of 22 patients, received PRP injections. Group B, consisting of 22 patients, was treated with a combination of conservative management approaches, including physiotherapy, oral analgesics (NSAIDs), and a counterforce brace. This randomization ensured that the baseline characteristics of the two groups were comparable, and any observed differences in outcomes could be attributed to the treatment methods rather than patient selection biases. The study was conducted in an open-label format without blinding, as the treatment and control groups required distinct interventions that could not be masked.

PRP preparation and injection protocol

For the PRP group, autologous PRP was prepared using a differential centrifugation technique. Approximately 15 mL of the patient’s venous blood was collected using a scalp vein catheter to minimize turbulence and was stored in three sterile tubes containing 0.9% sodium citrate as an anticoagulant. The blood was first subjected to centrifugation at 1500 rpm for 15 minutes. This initial centrifugation separated the blood components, allowing the upper plasma layer (platelet-poor plasma) to be discarded while the lower portion containing PRP was retained. The PRP was then further concentrated using a second spin, yielding 1 mL of highly concentrated PRP. Before injection, 1 mL of 1% lignocaine was mixed with the PRP solution for pain management. The PRP-lignocaine mixture was then injected into the most painful point on the lateral epicondyle, identified via palpation. The injection was administered under sterile conditions to minimize the risk of infection, and patients were advised to avoid strenuous activity involving the elbow for a minimum of two weeks post-injection.

Conservative management protocol

Patients in the conservative treatment group (Group B) were provided with a standardized protocol involving physiotherapy, oral NSAIDs, and the application of a counterforce brace. Physiotherapy consisted of a tailored regimen focusing on stretching and strengthening exercises targeting the extensor muscles of the forearm. Sessions were supervised by a licensed physiotherapist twice a week, and patients were encouraged to perform home-based exercises. For pain management, patients were prescribed etodolac 400 mg once daily for one week, to be taken after meals to reduce gastrointestinal side effects. Additionally, patients were given a counterforce brace to be worn during daily activities to alleviate stress on the lateral epicondyle. Patients were instructed on proper brace usage and were monitored during follow-up visits for adherence to the protocol.

Outcome measures

The primary outcome of the study was the change in pain and function scores, as measured by the Patient-Rated Tennis Elbow Evaluation (PRTEE) score [20]. The PRTEE is a validated 15-item questionnaire that assesses pain (0-50 points) and functional impairment (0-50 points) specific to lateral epicondylitis. The PRTEE score ranges from 0 (no pain or disability) to 100 (maximum pain and disability), with higher scores indicating greater impairment. PRTEE scores were recorded at baseline for all participants and then reassessed at four weeks, eight weeks, three months, and six months post-treatment. Follow-up evaluations were conducted in person during outpatient visits, and in cases where patients could not attend follow-ups, telephonic interviews were conducted to gather PRTEE data.

Statistical analysis

All data were entered into Microsoft Excel for initial data management and transferred to IBM SPSS Statistics for Windows, Version 23.0 (Released 2015; IBM Corp., Armonk, NY, USA) for statistical analysis. Descriptive statistics were calculated for all variables, with means and SDs reported for continuous variables. The independent t-test was employed to compare the mean PRTEE scores between the PRP and conservative treatment groups at each follow-up interval. A p-value of less than 0.05 was considered statistically significant. The improvement in pain and function scores within each group over time was also analyzed using paired t-tests. Additionally, the two groups documented and compared any complications or adverse events related to the treatments. The analysis aimed to determine whether PRP offers a statistically significant advantage over conservative management regarding long-term pain relief and functional improvement.

Ethical considerations

The ethical approval for this study was obtained from the Institutional Ethics Committee of Father Muller Medical College Hospital under the reference number FMMCIEC/CCM/200/2018. Before commencing the study, all procedures were reviewed and approved to ensure compliance with ethical standards. Participants were fully informed about the study’s objectives, potential risks, and benefits, and written informed consent was obtained from each patient before enrollment. This process ensured that participation was voluntary and that all patients understood their rights, including the option to withdraw from the study at any time without affecting their medical care. Throughout the study, patient confidentiality was maintained by anonymizing all personal data, which was securely stored and accessible only to authorized personnel. The study adhered to the ethical principles of beneficence, non-maleficence, autonomy, and justice, ensuring the well-being and safety of the participants while contributing to medical knowledge in the treatment of lateral epicondylitis.

## Results

The study included 44 patients with chronic lateral epicondylitis, divided equally into 22 in the PRP group and 22 in the conservative treatment group. The data was analyzed based on the PRTEE pain subscale, function subscale, and total score over six months. The analysis focuses on comparing the two groups’ short-term and long-term efficacy. The comparison of the PRTEE pain subscale between PRP and conservative treatment groups over the follow-up period is presented in Table 1.

**Table 1:** Comparison of PRTEE pain subscale between PRP and conservative groups PRP, platelet-rich plasma; PRTEE, Patient-Rated Tennis Elbow Evaluation

Timepoint	Conservative group (mean ± SD)	PRP group (mean ± SD)	t-value	p-value
Baseline	33.27 ± 5.53	33.77 ± 4.00	-0.344	0.733
Four weeks	32.32 ± 4.73	32.86 ± 4.13	-0.408	0.686
Eight weeks	31.86 ± 4.51	29.59 ± 3.74	1.821	0.076
Three months	32.23 ± 3.75	26.00 ± 3.55	5.657	<0.001
Six months	33.64 ± 3.63	19.55 ± 3.33	13.404	<0.001

The comparison of the PRTEE function subscale between PRP and conservative groups is detailed in Table 2.

**Table 2:** Comparison of PRTEE function subscale between PRP and conservative groups PRP, platelet-rich plasma; PRTEE, Patient-Rated Tennis Elbow Evaluation

Timepoint	Conservative group (mean ± SD)	PRP group (mean ± SD)	t-value	p-value
Baseline	30.73 ± 3.80	33.73 ± 3.18	-2.842	0.007
Four weeks	30.09 ± 4.15	32.41 ± 2.34	-2.281	0.028
Eight weeks	29.27 ± 4.21	29.86 ± 2.51	-0.565	0.575
Three months	29.59 ± 3.98	26.05 ± 2.38	3.583	0.001
Six months	30.73 ± 4.11	20.00 ± 3.19	9.672	<0.001

The total PRTEE scores for both groups are summarized in Table 3.

**Table 3:** Comparison of total PRTEE score between PRP and conservative groups PRP, platelet-rich plasma; PRTEE, Patient-Rated Tennis Elbow Evaluation

Timepoint	Conservative group (mean ± SD)	PRP group (mean ± SD)	t-value	p-value
Baseline	64.45 ± 9.03	68.41 ± 6.44	-1.672	0.102
Four weeks	62.86 ± 8.50	65.27 ± 5.44	-1.12	0.269
Eight weeks	61.18 ± 7.54	59.45 ± 5.28	0.88	0.384
Three months	61.82 ± 6.65	51.59 ± 4.55	5.955	<0.001
Six months	64.23 ± 6.67	40.00 ± 6.20	12.484	<0.001

Neither group reported any complications throughout the study, as presented in Table 4.

**Table 4:** Complication rate comparison between PRP and conservative groups PRP, platelet-rich plasma

Group	Complications (N, %)	Total (N, %)
Conservative (n = 22)	Nil (22, 100%)	22 (100%)
PRP (n = 22)	Nil (22, 100%)	22 (100%)

## Discussion

Lateral epicondylitis, or tennis elbow, is a prevalent musculoskeletal condition characterized by pain and tenderness over the lateral epicondyle of the humerus. The condition primarily affects the extensor muscles of the forearm, particularly the ECRB, leading to inflammation, microtears, and degenerative changes at the tendon insertion site. The prevalence of lateral epicondylitis ranges from 1% to 3% of the general population, with peak incidence occurring between the ages of 35 and 55 years, irrespective of gender or ethnicity [21]. The condition is frequently associated with repetitive wrist extension and overuse, often observed in occupations or sports that involve excessive manual labor or repetitive arm movements, such as tennis, hence the colloquial name [1]. The pathophysiology of lateral epicondylitis is not merely inflammatory but degenerative, as indicated by the absence of classic inflammatory cells on histopathological examination. Instead, the affected tendons show angiofibroblastic hyperplasia, marked by increased vascularity and disorganized collagen fibers [22]. This has led to the condition being described as tendinosis rather than tendinitis. Clinically, patients present with localized pain over the lateral epicondyle, exacerbated by wrist extension or gripping activities. Diagnostic tests such as Cozen’s and Mill’s tests are often positive, demonstrating tenderness and pain upon resisted wrist extension and passive wrist flexion [6].

Traditionally, the management of lateral epicondylitis has focused on conservative treatment modalities, including rest, physical therapy, bracing, and pharmacological interventions such as NSAIDs. Corticosteroid injections have also been widely used due to their anti-inflammatory properties. Still, their long-term efficacy remains controversial, with several studies suggesting an increased risk of recurrence and potential adverse effects such as tendon weakening and rupture [9,23]. More recently, attention has shifted toward regenerative therapies such as PRP injections, which utilize the patient’s platelets to stimulate healing by releasing growth factors [24]. PRP is a biological product derived from autologous blood in which the concentration of platelets is significantly higher than baseline levels. These platelets contain growth factors such as PDGF, TGF-β, VEGF, and EGF, all of which play a crucial role in tissue repair and regeneration [12]. By delivering these growth factors directly to the injury site, PRP is believed to accelerate the healing process, reduce inflammation, and promote tendon regeneration. Previous studies have shown that PRP can be particularly beneficial in chronic tendinopathies, including lateral epicondylitis, where conventional therapies often fail to provide long-term relief [17].

The use of PRP in lateral epicondylitis has gained popularity in recent years, with several studies suggesting its superiority over corticosteroids and other conservative treatments in terms of long-term pain reduction and functional improvement [25]. However, despite these promising results, the optimal role of PRP in managing lateral epicondylitis remains a subject of debate, with varying methodologies, follow-up durations, and patient outcomes reported in the literature [26]. Furthermore, while PRP appears to offer a safe and effective alternative to surgery for many patients, its high cost and lack of standardization in preparation and administration have limited its widespread adoption [27]. In light of these factors, this study aims to provide further evidence of the efficacy of PRP compared to traditional conservative treatments in managing chronic lateral epicondylitis. By assessing pain and functional outcomes over six months using the PRTEE score, this study seeks to determine whether PRP offers a significant advantage in terms of long-term efficacy and whether it should be considered a first-line treatment for patients with persistent lateral epicondylitis.

Limitations

This study has several limitations that should be acknowledged. Firstly, the sample size was relatively small (44 patients), which may limit the generalizability of the findings to the broader population. A larger sample size would provide more robust conclusions. Secondly, the study was an open-label observational study, meaning there was no blinding of the participants or investigators, which could introduce bias in assessing outcomes. Additionally, the study only included patients aged 18 to 60; thus, the results may not apply to younger or older populations. Another limitation is the relatively short follow-up period of six months. Although PRP demonstrated superior long-term efficacy in this period, longer-term studies are needed to assess the sustainability of these effects beyond six months. Lastly, while the PRTEE score was used as the primary outcome measure, the study did not include other potentially relevant outcomes, such as patient satisfaction or quality of life measures, which could provide a more comprehensive evaluation of the treatment’s impact.

## Conclusions

This study demonstrates that PRP injections offer superior long-term efficacy to conventional conservative treatments such as analgesics, physiotherapy, and counterforce bracing in managing lateral epicondylitis. While PRP and conservative treatments provided significant short-term relief, PRP showed sustained improvement in pain reduction and functional recovery over six months. The lack of complications in either group highlights the safety of both treatment modalities. However, the long-lasting benefits of PRP make it a more favorable option for patients seeking durable relief from chronic lateral epicondylitis. Further research, particularly larger, randomized controlled trials, is needed to confirm these findings and explore the long-term outcomes of PRP treatment beyond the six-month follow-up period.
